# The use of vaccines to control pathogen spread in pig populations

**DOI:** 10.1186/s40813-017-0053-6

**Published:** 2017-03-01

**Authors:** Nicolas Rose, Mathieu Andraud

**Affiliations:** 1 Anses-Laboratoire de Ploufragan-Plouzané, Swine Epidemiology and Welfare Research Unit, Po Box 53, F22440 Ploufragan, France; 2 Université Bretagne Loire, Rennes, France

**Keywords:** Vacccination, Infectious disesases, Reproduction number, Herd immunity, Pig populations

## Abstract

Vaccine efficacy has often been studied from the viewpoint of individual direct clinical protection. For several vaccines, a decrease in pathogen shedding in vaccinated animals has also been documented, which suggests that transmission between individuals has the potential to be reduced. In addition, vaccination induces an immune response in the host potentially decreasing susceptibility to infection in comparison with immunologically naïve animals. As a collective result of individual vaccinations, vaccine programmes generally have a wider impact on pathogen diffusion at the population scale. Beyond the individual protection conferred by mass vaccination campaigns, the indirect protection of non-immune individuals in contact with vaccinated ones also contributes to controlling pathogen spread at the population scale; a phenomenon known as herd immunity.

Pathogen spread within pig populations is strongly related to the required vaccine coverage at the population level and to pathogen characteristics in terms of diffusion (${R}_0$). Before setting up vaccination programmes, it is therefore necessary to have quantitative knowledge on vaccine efficacy as regards transmission reduction. These data can be obtained by carrying out experimental studies or observational protocols in real conditions. These quantitative data have mainly been estimated for major infectious diseases which have now been eradicated. A great gap in knowledge has however been identified for enzootic diseases which are daily impacting the swine sector as well as for the source of variation responsible for a decrease in vaccine efficacy as compared to assessments obtained in experimental conditions.

## Background

The main pursued effect of vaccines used in pig production and generally in animal production, is the individual direct protection. The main expected impact resides in a significant decrease of the clinical signs associated with the infection that the animals are vaccinated against. The decrease of pathogen shedding in vaccinated animals has also been documented for several vaccines. However, it is well established that the majority of vaccines used in human and veterinary medicine confer only partial protection, thus the risk of infection remains [1]. All vaccines used in pig production can be considered as such.

The effect of vaccination programmes at the population scale is the result of a collective impact of individual vaccinations on the transmission of the infection within the population. While the individual protection is the target objective of mass vaccination programmes (vaccination of the whole population), the global effect at the population scale also contributes indirectly to individual protection according to the “herd immunity” concept. The impact at the population scale of a vaccination programme depends on three main factors. First, the epidemiology of the pathogen and its transmission potential of the infectious agent, which is generally summarized by the basic reproduction number denoted ${R}_0$. This parameter corresponds to the average number of infected individuals produced by a typical infectious individual during its whole period of infectiousness in a fully susceptible and large population. Second, the ability of the vaccine to modify the contribution of the individual to the transmission of the infection within the population, which can be summarized by the vaccine efficacy as regards transmission. This characteristic combines the reduction of susceptibility to infection through a certain level of individual direct protection and the effect of vaccine on the reduction of infectivity (ability to transmit the pathogen). Susceptibility is defined by the probability of infection in vaccinated animals when in contact with shedding individuals while infectivity is represented by the probability of seeding new infections by shedding animals. For this latter parameter, vaccination can result in the decrease of the duration of pathogen transmission and/or of the quantity of pathogen shed by the host. Third, the vaccination programme and particularly the vaccine coverage within the population [2]. This last characteristic is linked to the reproduction number specific to the pathogen.

**Fig. 1:**
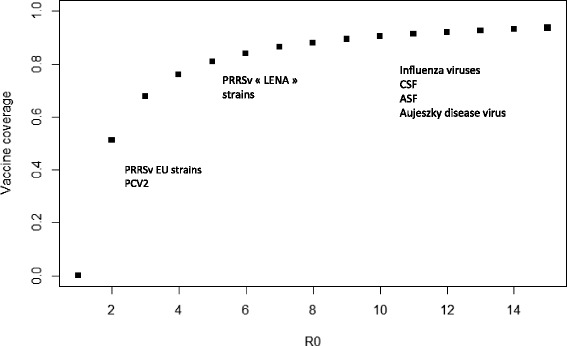
Relationships between the vaccine coverage within the population (proportion of the population to be immunized) and the ${R}_0$ value (particular case of vaccine conferring a “perfect” protection)

From a general point of view, the higher the ${R}_0$, the more difficult the pathogen to eradicate. For infection eradication, the effective reproduction number within the vaccinated population denoted $R$ must be brought below 1. It corresponds to the modification of pathogen transmission because of an efficient immunization of a proportion $P$ of pigs within the population. Indeed, let *x* = 1 − *P* correspond to the non-immune fraction in the population, then *R* = *R*_0_ *x* should get lower than 1 to reach eradication. Otherwise stated, the fraction of vaccinated population should exceed the critical vaccination coverage defined by ${P}_0=1-\frac{1}{R_0}$. This relation shows that eradication of a pathogen that has a high ${\mathrm{R}}_0$ requires extensive vaccine coverage (Fig. 1). Considering a full vaccine efficacy as regards the infectious agent, it follows that the fraction of the vaccinated population cannot get infected. In consequence, the number of infectious individuals is reduced, leading to a global decrease of the force of infection exerted on non-vaccinated animals. Each vaccinated individual contributes therefore to the global protection of the population which is known as “the herd immunity” [3].

This relationship between the vaccine coverage and ${R}_0$ allows the prediction of the benefit of vaccination in terms of reduction of the number of infectious individuals when the infectious process tends towards an equilibrium and according to the vaccine coverage which can be implemented considering economic and technical constraints (e.g. partial vaccination of the herd as the only affordable strategy). For an infectious agent such as Porcine Circovirus type 2 (PCV2) or a European strain of the Porcine Reproductive and Respiratory Syndrome virus (PRRSv), for which an average reproduction number of 5 has been estimated [4, 5], a vaccine coverage of 80% of the whole population should theoretically allow pathogen eradication. However, an effective vaccine coverage ${p}_E$ of 50% of the population would lead to a reduction of the number of infected individuals by 62% $\left({\mathrm{P}}_{\mathrm{e}},/,{\mathrm{P}}_0^{*},100\right)$.

One of the key factors to be accounted for in the field is that the protection conferred by the large majority of vaccines does not fully prevent infection. In this case, the critical threshold applies to the fraction of the population that should be immunized (according to the level of immunization conferred by the vaccine). A partial protection can reside in different causes: the vaccine can be able to reduce the susceptibility of pigs to the infection (decreasing the risk of getting infected); it can also decrease pathogen transmission once the pig is infected, or accelerate the elimination of the pathogen, hence reducing the duration of shedding. These vaccines, providing a limited protection from an epidemiological point of view, are generally called “leaky vaccines”. In this case, the critical vaccine coverage can be defined considering a reproduction number specific to the vaccinated population, ${R}_v$, parameter corresponding to the number of secondary cases produced by an infectious individual when the whole population is vaccinated. The critical threshold for vaccine coverage becomes therefore

$$ {V}_c=\frac{R_0-1}{R_0-{R}_v} $$

**Fig. 2:**
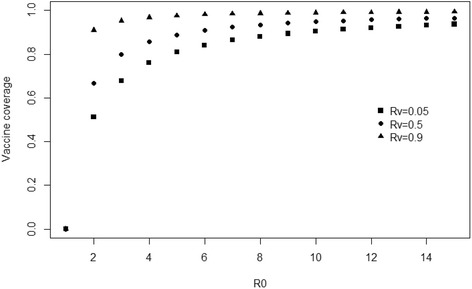
Evolution of the vaccine coverage (proportion of the population to be vaccinated) according to the basic reproduction number ${R}_0$ and for different values of ${R}_v$ (effective reproduction number when the whole population is vaccinated)

The eradication of the infection requires ${R}_v$ to be lower than 1. In Fig. 2, it appears that when a vaccine only induces partial protection to the animals, the eradication remains possible whenever ${R}_v$ <1, but would require the vaccination of the entire population when ${R}_v$ gets close to 1.

Building control programmes based on vaccine tools at the population level requires quantitative data on the ability of vaccines to decrease pathogen transmission within the target population. The following sections will aim at (i) describing the methodological approaches available for the estimation of the reproduction number in vaccinated population through the comparison of experimental and observational approaches, (ii) summarizing existing data for the different pig pathogens and their limitations. Applied consequences of these theoretical aspects are further confronted to the different vaccine practices and protocols usually set up in pig farms.

## Assessing the impact of a vaccine on pathogen transmission

In the objective of using a vaccine as an epidemiological tool for disease eradication or at least reducing the impact of an infection on the population, it is pivotal that the vaccine could significantly reduce the pathogen spread within the population. Classical studies based on the principle of vaccination followed by a challenge are not sufficient to produce information on pathogen transmission (how the pathogen spreads and at which rate), given the fact that these studies focus on infection characteristics at the individual level. It is therefore necessary to study how the pathogen spreads within a vaccinated population and which factors are likely to modify transmission. Pure qualitative studies describing the transmission of the pathogen to sentinel susceptible pigs do not inform on (i) the extent of the transmission process and (ii) the significance of observed differences in terms of transmission between vaccinated and non-vaccinated populations. These specific questions can only be addressed through quantitative estimation of the transmission potential between hosts. Both experimental studies in controlled conditions and observational studies in real conditions can be used to these ends. The specificity of studied infectious processes relies on dependent incidences of infections within different groups belonging to the same population, leading to non-linear dynamic infectious processes. In this specific case, non-linear mathematical models are useful to formulate hypothesis on mechanisms and types of transmission involved, understand the epidemiological processes and assess the potential efficacy of control measures [6].

### Experimental assessment: transmission trials

The efficacy of a vaccine regarding the reduction of transmission of an infectious agent must be assessed within homogeneous groups, which implies that all the pigs of a group, including sentinel ones, should be vaccinated, whereas pigs from the control group are not. Pigs belonging to the same group are assumed to be comparable, i.e. with a similar susceptibility to infection and a comparable infectiousness once infected. Moreover, they must move freely among the group to verify the hypothesis of homogenous contacts between pigs within groups. The transmission rate cannot be estimated properly through the involvement of pigs of heterogeneous statuses within experimental groups (e.g. mingling of fully susceptible and vaccinated animals within a group of vaccinated and further infected pigs). Vaccination generally reduces both the susceptibility to infection and the level of pathogen shedding once infected. Inoculating non vaccinated pigs within a group of vaccinated contact pigs can potentially lead to an extended shedding of the pathogen because non vaccinated and inoculated pigs are more infectious than vaccinated and further inoculated pigs. The other case corresponding to non-vaccinated pigs in contact with vaccinated and inoculated pigs can likely lead to an extended diffusion owing to a higher susceptibility to infection of contact pigs.

Three estimation methods are described in the literature to analyse such data. The principle of the statistical analysis of experimental transmission data can be based on the final state of the epidemic process observed at the end of the experiment [7]. The so-called « final size » algorithm (FS) consists in calculating probabilities of each possible final state [8]. Hence, for an experimental trial involving five susceptible individuals and five infectious pigs (*S*_0_ = 5, *I*_0_ = 5), the whole possible observations when the infectious process ends are (*S*, *I*) ∈ {(5, 0), (4, 0), (3, 0), (2, 0), (1, 0), (0, 0)}. To each of these states corresponds a probability dependent on the reproduction number ${R}_0$. This number is estimated by confronting the results of the experiment with the associated probabilities by the method of the maximum likelihood. The transient state algorithm (TS) relies on the other hand on a time-continuous version of the previous one and requires complex calculations because of its recursiveness and can therefore be used only for trials involving a limited number of animals [8].

As previous approaches, the estimation method based on the martingale formula proposed by Becker [9] requires to provide the initial number of susceptible and infectious, as well as the final state of the system, i.e. the number of remaining susceptible animals at the end of the experiment. This method is preferably used for large populations and therefore less adapted to experimental conditions. However, when all the replications of a transmission experiment result in the infection of all initially susceptible animals, estimations from the two first algorithms tend to infinity. Conversely, the martingale method allows an estimation of ${R}_0$ by evaluating the loss of infectiousness accounting for the remaining duration of shedding of infectious individuals after the infection of the last contact pig.

Another approach is based on the analysis of the kinetics of new infections occurring among sentinel pigs during the experiment. In this particular case, the estimation of the reproduction number is deduced from the independent estimation of the transmission rate, commonly denoted *β*, and from the duration of infectiousness 1/*σ*: *R*_0_ = *β*/*σ*. The approach is more dynamic and fully takes advantage of the longitudinal follow up of pigs during the experiment. Becker [9] proposed the use of generalized linear models (GLM) to estimate the transmission rate of an infectious agent within a population. This approach allows accounting for heterogeneity regarding both infectivity of infectious individuals and susceptibility for sentinel pigs. The limitations of the GLM approach are mainly due to the impossibility to account for a specific contact structure (e.g. within- and between-pen [10]). The statistical analysis of these data requires to write explicitly and to maximize the likelihood function linking the theoretical model and observed data. The duration of the infectious period is often assessed from virological and serological individual data using parametric survival models. These studies require a close individual follow-up of pigs with frequent monitoring of their status in order to determine precisely the number of new infections occurring between two sampling times. The protocol (number of pigs being inoculated, number of contact pigs, and interval between two samplings) must be determined according to the knowledge available on the transmission characteristics of the pathogen. For example a pathogen like influenza virus which can transmit very fast and efficiently on a very short period of time (5–6 days) would require a frequent sampling (daily or more) compared to other pathogens having a prolonged duration of shedding with a daily transmission rate which is much more lower (PRRS virus, PCV2).

### Observational approaches in real conditions

The main limitations of experimental studies are the potential extrapolation of results to field conditions. In experimental conditions, the pure infectious process is generally reproduced (without any co-infections, in small-size, homogeneous and low density population). For these reasons, estimations should be considered with care before transposing them to real situation with systematic co-infections. Consequently, observational studies carried out in real conditions constitute an ultimate step in vaccine evaluation. These studies are often based on a longitudinal follow-up of infection chains using a descriptor to assess the evolution of infectious individuals. Serology is generally used, because it is often the only option in terms of cost and feasibility for large scale studies in farms where large populations are sampled. However, the use of serology requires differentiating antibodies markers of a past exposure to the pathogen from potential antibodies induced by vaccination. This is only affordable when marked vaccines are used (deleted or allowing Differentiation between Infected and Vaccinated Animals [DIVA vaccines]) like Aujeszky’s disease vaccines. By studying observed infection chains based on seroconversion against gE protein within sow herds vaccinated every 4 months, only minor outbreaks were observed in a study carried out in The Netherlands, although susceptible animals were regularly introduced into the herds. In this study, the estimated reproduction number $R$ was 0.7 within these vaccinated populations [11]. The objective of another field study, also carried out in The Netherlands, was to compare two pseudorabies virus vaccination schemes in fattening pigs for a better control of the infection: a single injection around 14 weeks of age versus two injections at 11 and 15 weeks of age [12]. The authors, used observed seroconversion data against gE protein to estimate the reproduction number using the same approach as described for transmission experiments, i.e. a stochastic SIR model representing infection chains leading to a distribution of epidemic size at the end of the infectious process (frequency of pigs having anti gE antibodies at the end of fattening). By using these data, the authors showed that double vaccination decreased the reproduction number from 3.5 (single vaccination) to 1.5 (double) without leading to total eradication (R > 1). Discrepancies between field and experimental studies can reside in different factors such as the status of the animal at vaccination time, non-properly performed vaccination in field conditions, bioclimatic factors, other concurrent infections, etc.…

## Available data on quantification of the transmission of pathogens within a pig population

A summary of data available on the evaluation of the efficacy of vaccines as regards the reduction of transmission of main pathogens involved in pig production is presented in Table 1. The most numerous studies have been carried out on Aujeszky’s disease and classical swine fever because of the paramount importance of such data to set up eradication plans for these notifiable diseases. The majority of studies have been carried out in experimental conditions, except for Aujeszky’s disease for which several studies aiming at vaccine evaluation in field conditions have been set up at the end of the 90’s. Generally, a good agreement between results from different published studies has been observed showing either a good ability of vaccines to significantly decrease the transmission rate (with sometimes an expected reproduction number significantly lower than 1 in experimental conditions for Aujeszky’s disease, classical swine fever or PRRS); or the absence of effect of vaccination on transmission (*Mycoplasma* vaccines). Available data are very useful to incorporate parameter estimations to epidemiological models set up to assist decision makers in risk management [13]. However, differences between estimates obtained in experimental and real conditions suggest that the effect of vaccines can potentially be modulated by complex interactions between risk factors such as the viral strain, contact heterogeneity within the population, co-infections or husbandry practices. To further explore these complex interactions, more experimental studies would be required to evaluate the effect of vaccination on pathogen transmission in presence of co-infections, especially because numerous pathogens of importance for pig production are more involved in syndromes such as the Porcine Respiratory Disease Complex (PRDC) than in pure monofactorial diseases.

**Table 1:** Summary of main quantitative data on the evaluation of the effect of vaccines on the transmission of pig pathogens

	References	Number of publications	Main effect	Agreement between studies	Study category	Comments
Aujeszky’s disease	[6, 7, 11, 12, 15, 21–24]	9	Significant reduction of transmission	Good for experimental studies	Experimental and observational studies	Field studies indicate potential limitations of experimental estimations
Classical swine fever	[10, 16, 25–30]	8	Significant reduction of transmission	Good	Experimental studies	Variability of results according to vaccines, administration route and the delay between vaccination and challenge
*Mycoplasma Hyopneumoniae*	[31, 32]	2	No effect on transmission	Good	Experimental and field studies	
*Actinobacillus Pleuropneumoniae*	[33]	1	Decrease of infectiousness only	/	Experimental study	Separate evaluation of susceptibility and infectiousness
PRRSv	[17, 34, 35]	3	Significant reduction of transmission	Good	Experimental studies	Good results with vaccine and challenge strains belonging to the same genotype
Influenza	[36]	1	Significant reduction of transmission	/	Experimental study	Significant reduction of transmission even with an heterologous strain
PCV2	[5]	1	Significant reduction of transmission	/	Experimental study	Evaluation with a challenge strain heterologous (2b) to the vaccine strain (2a)

## Source of variation of vaccine efficacy and practical consequences on vaccination protocols

The theoretical efficacy of vaccines as regards pathogen transmission can be considerably decreased under the influence of several factors. The identification of these critical factors is of importance for an optimal use of vaccines in farms and the adaptation of the corresponding protocol. Moreover, the vaccination scheme in farms must be established considering the main goal of vaccination: is it targeted for piglet protection in their young age to reduce virus or bacteria propagation at that stage? Is the objective to limit pathogen circulation in the reproductive herd which constitutes a permanent reservoir and source of reinfection? Or is it to head towards a global eradication at the herd level?

We will examine consecutively in this part factors limiting vaccine efficacy as regards pathogen transmission to be considered in vaccination programmes definition as well as in the adaptation of protocols, consistently with the main goal which is pursued.

### Factors related to vaccine efficacy decrease

Specificities of vaccination in pig production reside in the immunization of young animals when they generally still have high levels of maternally derived antibodies against the pathogen when the latter is settled enzootically within the herd or when the breeders are regularly vaccinated against this pathogen. A recent experiment showed that the PRRSv post-vaccine humoral and cellular response were considerably affected in piglets having high levels of neutralizing maternally-derived antibodies at vaccination time [14]. Conversely to piglets without any neutralizing antibodies at vaccination time and evidencing a vaccine viremia as soon as 2 weeks post-vaccination, a rapid gamma interferon response and a fast seroconversion 7 days post-vaccination, piglets with high levels of neutralizing antibodies did not show any immune response following vaccination or a weak and delayed one (after 8 weeks post-vaccination). Similarly to results available for Aujeszky’s disease virus [15] or classical swine fever [16], other supplementary studies would be required to evaluate if this impaired immune response in vaccinated animals decreases the protection level of the vaccine and the theoretical impact it has on transmission reduction [17].

Beside this interference between maternally derived antibodies and vaccination, several diseases can be vertically transmitted and are susceptible to lead to viremic piglets at birth (PCV2, PRRSv for example). Vaccination against these infections is generally implemented at weaning, at 3 or 4 weeks of age, several piglets can therefore be vaccinated while being viremic in unstable herds (active circulation of the virus in the sow herd). Consequences of vaccinating a viremic piglet are not well known but it is likely that the immune system challenged by the concurrent infection would not be in the best situation to cope with the vaccine challenge and that the effect of vaccine would therefore be considerably reduced or abolished. Under certain circumstances (modified live vaccine), potential deleterious consequences should be also considered in case of simultaneous “co-infection” between the wild and the modified live vaccine strain possibly leading to reassortment events.

The influence of other circumstances on the quality of the post-vaccine immune response such as co-infections by different pathogens or the existence of bad environmental or rearing conditions, are not well known and would deserve to be better explored. Conversely, it has been shown that some feed contaminants (mycotoxins) could considerably decrease the quality of the immune response following vaccination such as the one implemented against PRRSv [18].

### Adaptation of vaccination scheme to the objective

Vaccination programmes are established in pig production according to the main goal which can be different according to the infectious agent and/or the sanitary status of the farm. Several vaccines only target breeders in order to protect against the reproduction disorders induced by the infection (case of porcine parvovirus for example). Other vaccines are both used in breeders and growing pigs. In this latter case, vaccination of young piglets aims at its direct protection against the exposure to targeted infectious agents during the growing part of the pigs (PRRSv, PCV2, *Mycoplasma* vaccinations). Vaccination of breeders is also often implemented to confer a certain level of specific immunity to piglets via the transfer of colostral immunity. Vaccination scheme consists in this case in boost injections at the end of gestation in sows to trigger the transfer of colostral immunity to the offspring (flu vaccination, PRRSv, PCV2, colibacillosis…). The objective in this case is a clinical protection of piglets during the lactation phase. However this passive immunity can also modify the propagation of pathogens in piglets young age. In the case of swine flu, it has been shown recently in a transmission experiment involving piglets born to vaccinated or non-vaccinated sows that the presence of maternally derived antibodies in piglets significantly decreased the virus transmission but not enough to block its propagation within the population. Moreover, the transmission intensity in this immunized population born to vaccinated sows was slowed down, increasing the total duration of the infectious process within the population and enhancing potentially the enzootic persistence of the virus in the farm [19, 20].

**Fig. 3:**
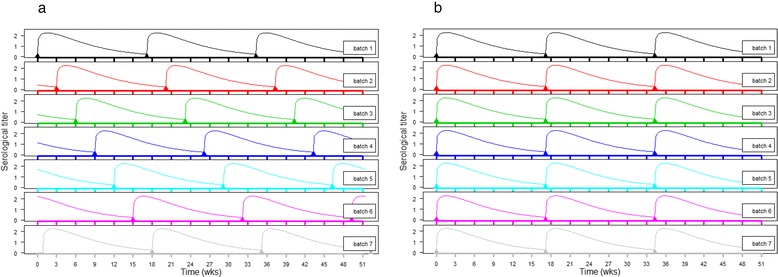
Theoretical comparison of « batch to batch » (**a**) and « mass » (**b**) vaccination from the vaccine coverage of the population point of view

When implementing vaccination programmes in farms, the consequences of the vaccination scheme in terms of pathogen spread should also be considered, especially if the objective must go beyond the only reduction of the clinical impact of the infection. The most commonly practiced vaccination scheme is the “batch to batch” programme consisting in the injection of the vaccine at a specific physiological status with a regular booster at every cycle to protect the sow and/or the offspring via colostral immunity. Because of batch-based rearing of the sow herd, all the batches are not synchronized in terms of immune response with an increase of immunity after each boost and a progressive waning of immunity in the following months. At a specific time, the level of immunity between batches is extremely heterogeneous and this heterogeneity is permanent with time, which suggests that the vaccination coverage at the population level never reaches 80% (Fig. 3). The so-called “mass” or “blanket” vaccination programmes resides in the principle of a boost vaccination on the overall population synchronized in time. This kind of protocol tends to provide a larger homogeneity in terms of immunity at the global population scale leading to a higher vaccination coverage with time. However, if the objective is also to protect piglets via colostral immunity, the situation in terms of expected level of passive immunity is much more heterogeneous than in the context of “batch to batch” vaccination.

## Conclusions

Vaccines can be used far beyond the only objective of conferring a clinical protection to the vaccinated population. They can also constitute powerful tools for disease control in animal populations as demonstrated in the past for Aujeszky’s disease or classical swine fever. However, this efficacy must be evaluated through specific experimental or observational studies which can provide quantitative estimates of the impact of vaccination on pathogen spread within a population. The examination of available quantitative data on vaccine efficacy towards transmission evidenced an important lack of information for several pathogens of importance in pig production with only few experimental studies. Moreover, pure experimental assessment can represent important limitations in data extrapolation to field conditions and several discrepancies have been shown between experimental and field assessment such as for Aujeszky’s disease for example. Several variation sources of vaccine expected efficacy have been shown but the pivotal role of interactions between pathogens in case of co-infections still need to be examined in details. Vaccination practices in veterinary medicine should be more and more considered at the population scale and accounting for the main objective pursued. If experimental studies cannot fully answer to all these questions, this is because of multiple factors potentially modifying the efficacy of vaccines in real conditions related to herd characteristics, biosecurity and husbandry practices. Although the analysis of experimental or field data provides important general insights on the infectious processes, herd-specific conditions have to be considered to adapt control measures to each specific epidemiological situation.

## Abbreviations

DIVA: Differentiating Infected from Vaccinated Animals
GLM: Generalized linear model
PCV2: Porcine Circovirus type 2
PRDC: Porcine Respiratory Disease Complex
PRRSv: Porcine Reproductive and Respiratory Syndrome Virus
R: Effective reproduction number
R_0_: Basic reproduction number
R_v_: Reproduction number within a fully vaccinated population
V_c_: Vaccination coverage
